# A stochastic simulation model of African swine fever transmission in domestic pig farms in the Red River Delta region in Vietnam

**DOI:** 10.1111/tbed.13802

**Published:** 2020-08-29

**Authors:** Hu Suk Lee, Krishna K. Thakur, Vuong Nghia Bui, Thanh Long Pham, Anh Ngoc Bui, Tung Duy Dao, Vu Thi Thanh, Barbara Wieland

**Affiliations:** ^1^ International Livestock Research Institute (ILRI) Hanoi Vietnam; ^2^ Department of Health Management Atlantic Veterinary College University of Prince Edward Island Charlottetown PEI Canada; ^3^ National Institute of Veterinary Research Hanoi Vietnam; ^4^ Department of Animal Health Epidemiology Division Hanoi Vietnam; ^5^ International Livestock Research Institute (ILRI) Addis Ababa Ethiopia

**Keywords:** African swine fever, epidemiology, simulation model, transmission, Vietnam

## Abstract

The main objectives of this study were to model various scenarios of African swine fever (ASF) virus transmission among farms in Vietnam and to evaluate the impact of control strategies using North American Animal Disease Spread Model (NAADSM). A total of 7,882 pig farms in the Red River Delta (RRD) region were obtained from the General Statistics Office, and then, random points corresponding to the number of farms in each province were generated as exact farm locations were not available. A total of 10 models were developed, including movement control scenarios. In addition, we conducted sensitivity analysis to assess the impact of indirect contact transmission probability (TP). Overall, the indirect contact exhibited an important role in transmitting the ASF virus. In order to minimize ASF transmission between farms, we found that movement restriction needed to reach a certain level (approximately between 50% and 75%) and that the restriction had to be applied in a timely manner. This study offers valuable insight into how ASF virus can be transmitted via direct and indirect contact and controlled among farms under the various simulation scenarios. Our results suggest that the enforcement of movement restriction was an effective control measure as soon as the outbreaks were reported. In addition, this study provided evidence that high standards of biosecurity can contribute to the reduction of disease spread.

## 1 INTRODUCTION

African swine fever (ASF) is a highly contagious virus and classified as a notifiable disease by the World Organization for Animal health (OIE) (Tulman, Delhon, Ku, & Rock, 2009). It is a double‐stranded DNA virus of the *Asfarviridae* family, genus *Asfivirus* (Dixon et al., 2005). The disease causes acute haemorrhagic fever with mortality of up to 100% depending on the virulence of the isolate, dose and route of exposure to the virus (Costard, Mur, Lubroth, Sanchez‐Vizcaino, & Pfeiffer, 2013). Pigs are infected via contact with infected animals (including free‐ranging and wild pigs), fomites, premises, vehicles, clothes, consumption of contaminated feed and bites of infected ticks (OIE, 2020a). The disease can have serious economic consequences, through reduced international trade and a decrease in pig populations, which can in turn pose a huge threat to food security (Blome, Gabriel, & Beer, 2013). The disease is endemic in sub‐Saharan African countries, Caucasus, Eastern Europe and Baltic countries (OIE, 2020b). In Asia, the first outbreak was confirmed in northeastern China in August 2018 (Zhou et al., 2018), and then, the virus rapidly spread to other Asian countries (Dixon, Sun, & Roberts, 2019; FAO, 2020). In Vietnam, the first ASF outbreak was reported in February 2019 in backyard pig farms in Hung Yen province (Van Phan Le et al., 2019). Since then, ASF outbreaks have been reported in all 63 provinces, resulting in approximately 6 million pigs (20% of pig production) that have been either culled or killed by the disease (FAO, 2020). As of May 2020, outbreaks have not been reported for more than 30 days in 35 of the 63 provinces.

The use of simulation models for infectious diseases is an important tool for decision‐makers to evaluate the impact of outbreaks and to identify cost‐effective control strategies (e.g. vaccination, movement control and depopulation) (Francis, Klotz, Harvey, & Stacey, 2010; Keeling, 2005; Morris, Wilesmith, Stern, Sanson, & Stevenson, 2001). The North American Animal Disease Spread Model (NAADSM) is a computer software used to develop simulation models of the spread of highly infectious animal diseases (Harvey et al., 2007). It provides a flexible framework with user‐established parameters to define the disease spread by direct, indirect contact, airborne and local spread as well as assess the impact of various control measures.

In Vietnam, the first study using NAADSM explored porcine reproductive and respiratory syndrome (PRRS) transmission via direct and indirect contacts across different farm types (Lee et al., 2019). With the ongoing outbreak and impact of ASF on the pig sector, the main objectives of this study were to model various scenarios of ASF virus transmission among farms and to evaluate the impact of control strategies using NAADSM.

## 2 MATERIALS AND METHODS

### 2.1 Study location and population

**FIGURE 1:**
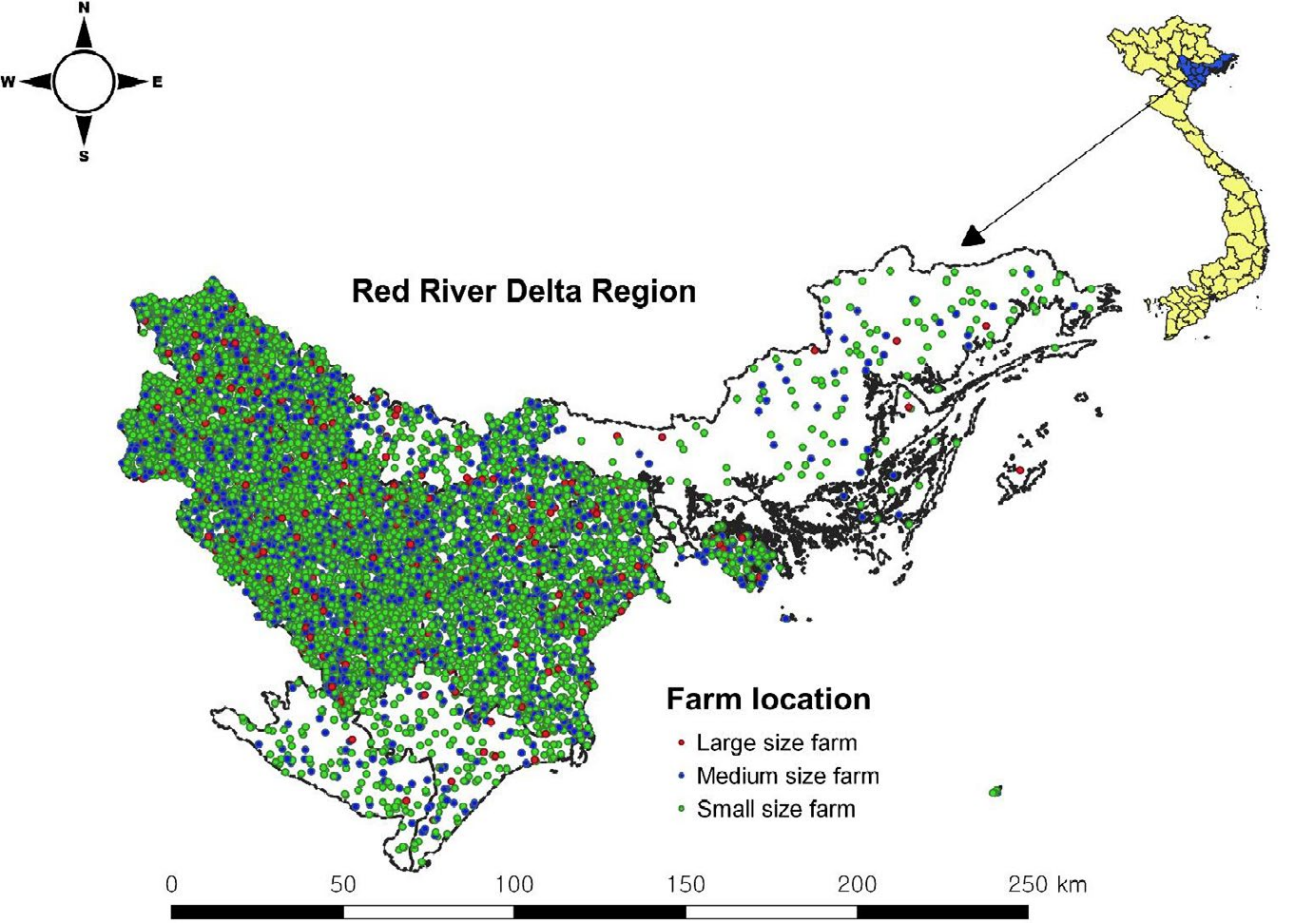
Spatial distribution of three pig farm types in the Red River delta region [Colour figure can be viewed at wileyonlinelibrary.com]

The Red River Delta (RRD) region is the smallest region in Vietnam, located in the north (Figure 1), yet it has the highest concentration of human population (22 million) in the country (GSO, 2020). It also has the largest pig population (7.2 million), which accounts for 28%–29% of the pig population in Vietnam (GSO, 2018). In order to develop the ASF transmission models, farm locations and characteristics were necessary. The number of livestock farms at provincial level was obtained from the General Statistics Office of Vietnam. As exact farm locations were not available, random points corresponding to the number of farms in each province were generated using QGIS (Quantum GIS development 2012, QGIS version 3.12.2) (Figure 1). The coordinates were then extracted and imported into NAADSM. A total of 7,882 farms were recorded and used for the simulation model. These farms were categorized into 3 production types: a) small (<100 pigs), b) medium (≥100 pigs) and c) large (>1,000 pigs) (Nga, Ninh, Van Hung, & Lapar, 2014). The proportion of each production type was 70% (a), 25% (b) and 5% (c), respectively (Lapar, Binh, & Ehui, 2003), Table 1.

**TABLE 1:** Model parameters used for simulation model between pig farm spread of ASF virus in the Red River Delta region, Vietnam

Parameters	Value	Reference
Total farms (*n*)	7,882	General statistics office of Vietnam
Small	5,499 (69.77%)	
Medium	1,989 (25.23%)	
Large	394 (5.00%)	
Transmission probability		
Direct contact	0.6^a^	(Guinat et al., 2016)
Indirect contact to small/medium farms	0.6	Contaminated products (e.g. swill) and vehicle movements are the main source of infection which was determined as the same as direct contact
Indirect contact to large farms	0.006	Due to high biosecurity
Infectious duration		
Small	52 weeks	Assumptions
Medium	10–12 weeks	Assumptions
Large	4 weeks	Assumptions
Contact distances between farms (km)	BetaPERT (0.5, 30, 300)	

^a^ The median value was used.

### 2.2 Model parameters

The NAADSM requires three key parameters: (a) transmission probability (TP) related to each contact (it is a probability between 0 and 1, and representing the likelihood of the contact herd will become infected given the exposure to an infected herd); (b) distance distribution associated with contact between farms; and (c) mean contact rates (estimated number of contacts per week) (Harvey et al., 2007). These parameters were estimated based on previous studies (Guinat et al., 2016; Lee et al., 2019) and assumptions (expert opinions) (Table 1). Transmission of ASF in Vietnam is mainly through indirect contact (e.g. swill feeding and human/vehicle movements). Therefore, TPs for direct and indirect contacts were considered as the same value (0.6) (expert opinions) for small and medium farms, whereas large farms were parametrized to have the value of 0.006 for indirect contact and 0.6 for direct contact TP due to comparatively higher levels of biosecurity (Table 1). These values were internally discussed among local experts. We assumed that the different infectious durations were determined by farm type (small farm: 52 weeks; medium farm: 10–12 weeks; and large farm: 4 weeks) as a result of different levels of biosecurity. Because, we assumed that a continuous flow (CF) system was used in small farms as they are replacing pigs continuously from different sources (with unknown disease status), while the all‐in‐all‐out system (AIAO) was followed in large and some medium farms where these farms introduce new pigs in batches and mostly from farms with high biosecurity and known infection status. Our estimates of infectious duration for the three farm types were based on these considerations, where large and medium farms were allowed to remain infectious for a relatively shorter duration. In contrast, small farms due to the continuous reintroduction of animals were allowed to remain infectious for the entire simulation duration.

A PERT distribution was defined for contact distances between farms, with a minimum of 0.5 km, a most likely value of 30 km and a maximum of 300 km (Table 1). The weekly mean contact rates (following a Poisson distribution) between different farm types were obtained from a previous study and plugged into simulation models (Lee et al., 2019) (Table 2).

**TABLE 2:** Contact structure of pig farms used by production type for simulation model

Contact groups (Source–Destination)	Mean contact rate/week (Lee et al., 2019)	
	Direct	Indirect
Small farms → Small farms	Poisson 0.072	Poisson 0.282
Small farms → Medium farms	‐	Poisson 0.282
Medium farms → Small farms	Poisson 0.072	Poisson 0.282
Medium farms → Medium farms	Poisson 0.073	Poisson 0.271
Medium farms → Large farms	‐	Poisson 3.5
Large farms → Medium farms	Poisson 0.073	Poisson 0.271
Large farms → Large farms	‐	Poisson 3.5

### 2.3 Simulation model structure and sensitivity analysis

**FIGURE 2:**
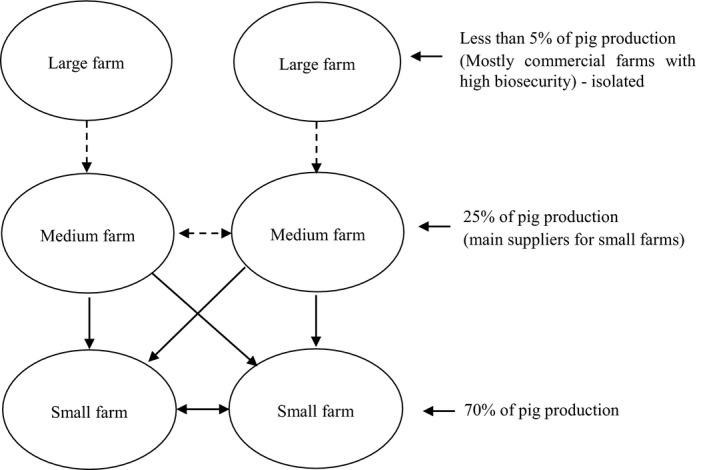
The simple diagram of network movement structure in Vietnam (dash arrow: rare movement)

In Vietnam, most large farms are commercialized and contract farms, while medium farms are mainly suppliers and have high connectivity to small farms (Figure 2). It is very rare that pigs from small farms move to other sized farms locally. We assumed that none of the pigs had resistance to ASF virus. If a single pig became infected, then the whole farm was considered to be infectious. The baseline scenario was that one medium farm was infected and the same farm‐initiated infection in the following iterations. We assumed that the rest of the farms were susceptible at the beginning of the simulation and remained infectious until the end of the study period for small farms or remained infectious for a specified period for medium and large farms and became susceptible again (Table 1). Especially, medium and large farms were allowed to be infected multiple times during the simulation.

The model was run over 500 iterations for 52 weeks, which was long enough to cover at least one complete pig production cycle (6–8 months in Vietnam). Since there is no vaccine for ASF, we evaluated the effectiveness of movement control on contact rates by 25%, 50%, 75% and 100% reduction, which was applied to both direct and indirect contact rates. It was hypothesized that movement restrictions were imposed for the baseline scenario within 4 weeks of detection of outbreaks. We conducted sensitivity analysis to assess the impact of infectious duration for small farms (basic: 52 weeks) and indirect contact TP for the small/medium farms (basic: 0.6) by −25%, −50% and −75%, respectively. In addition, the timing of movement restriction was imposed at 2 weeks, 6 weeks and 8 weeks after detection, respectively, under the 25% and 50% movement restriction scenarios.

## 3 RESULTS

The baseline scenarios (A1: including both direct and indirect contacts) showed that a total of 7,640 (5 and 95 percentiles: 6,729–7,790) median farms were infected, while A2 (only including indirect contact) presented a slightly lower median number of infected farms (7,544, 5 and 95 percentiles: 5,890–7,685) (Table 3). The epidemic peak was reached the earliest in scenarios (week 33) of A1 and A2 compared to A3 (no contact to large farms). Overall, the indirect contact exhibited an important role in transmitting the ASF virus. We evaluated the impact of movement restriction strategies on the number of infected farms. The MC1 scenario (25% reduction of contact rates) showed that the medium number of infected farms reduced by 19.28% compared to the baseline scenario (A1), while the large farms had a higher reduction of cases (−28.87%) compared to the small (−24.79%) and medium farms (−3.79%) (Table 4). The number of median infected farms for scenario MC3 (75% reduction of contact rates) dramatically decreased by 99.96% compared to the scenario A1, which was considered to be a very effective option. Overall, we found that the medium number of infected farms decreased as the contact rates reduced. Interestingly, in some iterations, the virus did not spread beyond the index farm under the M1, M2 and M3 scenarios. The proportion of such iterations with no infection spread for scenarios M1‐3 were 8.9%, 26.7% and 39.7% of the total 500 simulated iterations, respectively. We found that 4.9% and 9.2% of total infected large and medium farms had more than one outbreak, respectively, during the simulation.

**TABLE 3:** Median number of infected pig farms and time required to reach the peak epidemic under assumptions of various direct and indirect contacts

Scenario	Contact information	No. of mean infected farms (5 and 95 percentiles)				Week to peak epidemic
		Overall	Small	Medium	Large	
A1	Direct and indirect contact	7,640 (6,729–7,790)	5,231 (4,433–5,358)	2,084 (2,018–2,102)	324 (276–345)	33
A2	Indirect contact	7,544 (5,890–7,686)	5,144 (3,722–5,279)	2,079 (1,945–2,100)	323 (238–348)	33
A3	Indirect contact (no contact to large farms)	7,153 (5,801–7371)	5,074 (3,831–5,278)	2,077 (1,970–2,101)	0	35

**TABLE 4:** Median number of infected pig farms under the different movement restrictions on the contact rates

Scenario	Movement control	No. of mean infected farms (5 and 95 percentiles)				% change in the number of median infected farm
		Overall	Small	Medium	Large	
A1	Baseline	7,640 (6,729–7,790)	5,231 (4,433–5,358)	2,084 (2,018–2,102)	324 (276–345)	NA
MC1	25%	6,171 (0–6,950)	3,934 (0–4,615)	2,005 (0–2,075)	231 (0–273)	−19.23%
MC2	50%	1,231 (0–3,071)	639 (0–1,679)	560 (0–1,298)	35 (0–93)	−83.89%
MC3	75%	30 (0–159)	15 (0–79)	14 (0–77)	1 (0–4)	−99.62%
MC4	100%	1 (0–3)	0 (0–2)	0 (0–2)	0 (0–1)	−99.99%

The sensitivity analysis was implemented to evaluate on infectious duration (small farms; 52, 39, 26 and 13 weeks), TPs for indirect contact (small and medium farms), movement restrictions and the timing of restrictions compared to the baseline scenario. We found that the infectious duration did not have an impact on the results (less than 2% increase, not shown). The number of medium infected farms for scenario IC3 had a sharp reduction by 99.99% compared to the baseline scenario (A1) (Table 5). Overall, we found that the median number of infected farms had reduced as the TPs for indirect rates decreased. The timing of control measure did not have much impact on the median number of infected farms compared to the baseline (MC1) (Table 6). In addition, in the movement restriction scenario by 50%, it showed that TC6 had the relatively large increase in the median number of infected farms (30.38%). Overall, in order to minimize ASF transmission between farms, we found that movement restriction needed to reach a certain level (approximately between 50% and 75%) and that the restriction had to be applied in a timely manner.

**TABLE 5:** Sensitivity analysis of the median epidemic size of simulated ASF outbreaks to indirect contact transmission probability in a population of 7,882 pig farms

Scenarios	Parameters		±% change of parameters		Epidemic size median (5 and 95 percentile)	% change in median outcome compared to baseline
	DC Transmission probability	IC Transmission probability	DC Transmission probability	IC Transmission probability		
Baseline	0.6	0.6	N/A	N/A	7,640 (6,729–7,790)	N/A
IC1	0.6	0.45	N/A	−25%	6,201 (1,699–7,015)	−18.84%
IC2	0.6	0.3	N/A	−50%	1,106 (0–3,152)	−85.52%
IC3	0.6	0.15	N/A	−75%	10 (0–152)	−99.99%

Abbreviation: DC, direct contact; IC (small and medium farms): indirect contact.

**TABLE 6:** Sensitivity analysis of the median epidemic size of simulated ASF outbreaks to timing of movement restriction under the reduction of contact rate by 25% and 50%

Scenarios	Parameters		±% change of parameters		Epidemic size median (5 and 95 percentile)	% change in median outcome compared to baseline
	Movement restriction	Timing	Movement restriction	Timing		
MC1	25%	4 weeks	N/A	N/A	6,171 (0–6,950)	N/A
TC1	25%	2 weeks	N/A	−50%	6,145 (16–6,913)	−0.42%
TC2	25%	6 weeks	N/A	50%	6,172 (611–6,980)	0.02%
TC3	25%	8 weeks	N/A	100%	6,276 (1,379–7,026)	1.70%
MC2	50%	4 weeks	N/A	N/A	1,231 (0–3,071)	N/A
TC4	50%	2 weeks	N/A	−50%	1,180 (0–2,933)	−4.14%
TC5	50%	6 weeks	N/A	50%	1,440 (0–3313)	16.98%
TC6	50%	8 weeks	N/A	100%	1605 (0–3477)	30.38%

## 4 DISCUSSION

This was the first study in Vietnam to assess the transmission of ASF virus among swine farms using NAADSM. The weekly mean contact rates by farm types were obtained from the previous study in Vietnam (Lee et al., 2019), which made our model more realistic in terms of applicability to local farms. In the model, indirect contact had a predominant role in the transmission of the ASF virus between farms. It has been suggested that the various means of indirect contact (e.g. swill feeding, human/transport‐associated routes and improper disinfection) account for 70%–80% of the transmission of ASF virus among farms in Vietnam (DAH, 2020). In fact, it is still a common practice to give swill feeding in small pig holders in Vietnam even after ASF outbreaks have occurred. In addition, it is well known that wild boars and soft tickets could be the main source of infection in other countries (Galindo & Alonso, 2017; Thomson, 1985). In Asia, infected wild boars have been reported in China and South Korea (FAO, 2020; Li et al., 2019). However, no studies have been conducted to assess the roles of wild boars and soft tickets for virus spread in Vietnam. Therefore, further thorough investigation is necessary to identify the transmission route of ASF virus at farm level.

In terms of control measures, we evaluated the impact of movement restriction on the number of infected farms compared to the baseline scenario. Our scenarios suggest that strict movement control should be imposed to prevent the onwards disease transmission, which is consistent with other studies (Nielen, Jalvingh, Meuwissen, Horst, & Dijkhuizen, 1999; Turner, Bowers, & Baylis, 2012). In addition, the TC1‐6 scenarios showed that the timing of movement control was not an important factor under the loosen movement restriction scenarios. In Vietnam, when the first outbreak was confirmed in February 2019, the prime minister issued a directive to all provinces to apply all necessary control measures, including strict movement restrictions of pigs and pig products from infected provinces to other parts of the country, especially the south. In spite of this, within 8–9 months, ASF outbreaks were reported in all provinces. Monitoring of movement was poor mainly due to lack of management capacity and the low density of quarantine checkpoints, which were only set up at national highways and major routes across the provinces. Pig traders used alternative routes to avoid the quarantine checkpoints. In addition, some farmers who thought the ASF virus was a zoonotic disease urgently sold pigs through illegal means during the outbreaks, especially during the Tet holiday period (Vietnamese New Year in February).

Our simulation models showed that a decrease of indirect contact for TP resulted in a reduction of the number of infected farms when it reached a certain low level; otherwise, it was not effective. The main implication was that strict enforcement of high levels of biosecurity measures was the effective way to prevent the introduction of disease into pig farms. In Vietnam, poor biosecurity in small‐ and medium‐scale farms has already been identified as the main risk factor for disease transmission (Lee et al., 2020). Indeed, the absence of disinfection mattresses, no or rare use of protective boots and clothes, irregular disinfection of farm premises and the use of left‐over food for feedings are very common. One study showed that the biosecurity scores (it evaluates both external biosecurity [reduce the introduction of diseases] and internal biosecurity [reduce the spread of diseases]) in pig farms were between 53.68% and 55.05% based on percentage grade (0%–100%) (Tuan, Dewulf, Postma, Cuc, & Dinh, 2019). It is therefore very important to establish regular training programmes to educate farmers on biosecurity practices.

However, we acknowledge that in the absence of available data, the indirect contact TP, for small‐ and medium‐scale farms (0.6), is used in the baseline scenario and other movement control scenarios, which was based on our assumption owing to the above considerations. Our indirect contact TP is larger than the probabilities used for this parameter in ASF spread models in different jurisdictions. Our sensitivity analysis supported our assumption that indirect contact had a larger role in ASF spread in Vietnam as smaller indirect contact TP had resulted in nominal spread of the virus, contrary to what had been observed during the initial ASF outbreaks (much rapid/wider spread) in Vietnam. Although our simulation provided some guidance on the probable range of this probability, the uncertainty in this parameter estimate was still not resolved, and future field studies may help to provide better estimates.

It was assumed that the ASF virus was introduced from China as the virus strain was 100% identical to China strains (Van Phan Le et al., 2019). The most probable route of transmission was through the importation of pork products through illegal channels from China to Vietnam. In fact, the first outbreak was detected in the northern part of Vietnam where illegal animal/meat product movements are commonly reported (FAO, 2018). From then on, it was likely that the virus had spread to central and south provinces from the RDD (north part of Vietnam). This pattern was very similar when highly pathogenic porcine reproductive and respiratory syndrome (HP‐PRRS) outbreaks occurred in 2007 (Metwally et al., 2010). Another study also found similar pig movement patterns (Baudon et al., 2017).

According to local policy, all pigs in infected small farms must be culled, while neighbouring pigs in small farms without any suspected infections are not culled but are closely monitored until the outbreak is resolved. After culling, farmers receive different compensation rates based on the weight of breeding sows/boars and other pigs. However, farmers have a tendency to hide or postpone the reporting of suspicious cases (dead or alive pigs) to authorities. There are several reasons for this. Firstly, the symptoms of ASF are not clearly distinguishable from other diseases (e.g. Classical swine fever), particularly at the early clinical stages. Most smallholder farmers also do not fully vaccinate their pigs; therefore, it is easy for them to assume that their pigs are ill or have died from diseases other than ASF. Secondly, farmers fear the loss of all their pigs once a single pig becomes infected with ASF virus, because all pigs kept in the same pen should be depopulated. Thirdly, compensation procedures are complicated, with low compensation rates and long waiting periods, varying from several months to years depending on the availability of funds from the local authority. Lastly, farmers with infected pigs are concerned about the negative impact on their farm's reputation within their local community.

This model assumed that the whole farm became infectious if one pig was infected within the herd, which is realistic because of the highly contagious nature of the virus. There is a low probability that the ASF virus would fade out without onward transmission to other pigs in the farms. Some studies have suggested that R0 of ASF virus was estimated more than 1 in other countries (Barongo et al., 2015; Guinat et al., 2016; Iglesias et al., 2016). The limitation of this study was that individual pig farms have their own contact structures. However, in the model, contacts with the pre‐determined combination of farm types were random, within the given distance distribution, which may have resulted in an over estimation of outbreak size (especially in large farm types). Our model showed that quite a number of large farms were infected, whereas in reality, only few large farms were reported to have been affected up to now. The local‐area spread was not considered in the model. In particular, water, air and rodents may also contribute to the introduction and spread of the disease, given the fact that keeping pigs in open housing systems is a common practice of pig farms in Vietnam. Some studies have suggested that the ASF virus transmission was associated with aerosols, pest and rodent (de Carvalho Ferreira, Weesendorp, Quak, Stegeman, & Loeffen, 2013; Fasina et al., 2012; Olesen et al., 2017). In addition, our results may be influenced by the local‐area spread if some farms are in closer proximity. Therefore, in order to evaluate the impact of randomly created farm locations using QGIS, two more data were generated to make a comparison. We found that their impacts on the outcomes were negligible. In the study, the actual number of farms (especially, smallholders) in the RDD region may be much higher than the national data. Indeed, it is not easy to identify the number of smallholder farms (e.g. less than 10 pigs) in very remote rural and high mountainous areas unless farmers are willing to register. Therefore, it may be possible that the transmission of ASF virus was much faster than it was in our models.

This study offers valuable insight into how ASF virus can be transmitted via direct and indirect contact and controlled among farms under the various simulation scenarios. Our results suggest that the enforcement of movement restriction was an effective control measure as soon as the outbreaks were reported. In addition, this study provided evidence that high standards of biosecurity can contribute to the reduction of disease spread. This simulation model can be applied to other regions or countries with modified parameters. In addition, it may be useful for assessing the cost‐effective infection control and prevention strategies in the Vietnamese context through running the ‘what‐if’ scenarios related to ASF virus transmission.

## CONFLICT OF INTEREST

The authors declare that they have no competing interests.

## AUTHOR CONTRIBUTIONS

H.S.L, K.K.T and B.W. designed research. H.S.L performed research. H.S.L and K.K.T. analysed data. H.S.L, K.K.T. VNB, TLP, ANB, TDD, VTT and B.W. wrote the paper.

## ETHICAL APPROVAL

Ethics approval was not required for this study as any sample collection or questionnaires from animal/human has not been gathered.

## Data Availability

All datasets supporting our findings are available from the corresponding author on reasonable request.
